# Development and validation of a prediction model for in-hospital mortality in intensive care unit patients with cirrhosis and sepsis: a multicentre retrospective cohort study

**DOI:** 10.3389/fmed.2026.1759988

**Published:** 2026-02-09

**Authors:** Zhikun Xu, Qinhua Yang, Dongting Peng, Yichun Jiang, Yijing Su, Boru Wu, Zhiming Chen, Jiayang Huang, Xueyan Liu

**Affiliations:** 1 Department of Critical Care Medicine, Shenzhen People's Hospital (The First Affiliated Hospital, Southern University of Science and Technology), Shenzhen, China; 2 Department of Critical Care Medicine, Shenzhen People's Hospital (The Second Clinical Medical College, Jinan University), Shenzhen, China; 3 Department of Gastroenterology, Shenzhen Luohu People's Hospital, The Third Affiliated Hospital of Shenzhen University, Shenzhen, China; 4 Department of Anesthesiology, Shenzhen People's Hospital (The First Affiliated Hospital, Southern University of Science and Technology), Shenzhen, China; 5 Department of Anesthesiology, Shenzhen People's Hospital (The Second Clinical Medical College, Jinan University), Shenzhen, China; 6 Department of Pharmacy, Shenzhen People's Hospital (The First Affiliated Hospital, Southern University of Science and Technology), Shenzhen, China; 7 Department of Pharmacy, Shenzhen People's Hospital (The Second Clinical Medical College, Jinan University), Shenzhen, China

**Keywords:** cirrhosis, sepsis, mortality, prediction model, nomogram, intensive care unit

## Abstract

**Introduction:**

This study aimed to develop and validate a novel prediction model for in-hospital mortality among patients with cirrhosis and sepsis admitted to the intensive care unit (ICU).

**Methods:**

Data were obtained from three multicentre databases: the Medical Information Mart for Intensive Care IV (MIMIC-IV v3.1), the eICU Collaborative Research Database (eICU-CRD v2.0), and the Shenzhen People's Hospital ICU (SZPH-ICU). The MIMIC-IV cohort was chronologically divided into a training set (2008–2016) and a temporal validation set (2017–2022), whereas the eICU-CRD and SZPH-ICU cohorts were used for external validation. Variable selection was performed using the least absolute shrinkage and selection operator (LASSO) regression. A multivariable logistic regression model was constructed and visualized as a nomogram. Model performance was assessed using the area under the receiver operating characteristic curve (AUROC), Brier score, calibration plots, and decision curve analysis. A web-based calculator was developed to facilitate clinical implementation.

**Results:**

A total of 2,052 adult ICU patients with cirrhosis and sepsis from the MIMIC-IV database were included. The training cohort (2008–2016; *n* = 1,328) had a 24.0% in-hospital mortality rate, whereas the temporal validation cohort (2017–2022; *n* = 724) had a 35.9% in-hospital mortality rate. In the external validation cohorts, in-hospital mortality was 25.9% in the eICU-CRD (*n* = 657) and 38.2% in the SZPH-ICU (*n* = 131). The final model comprised 13 predictors: age, respiratory rate, body temperature, oxygen saturation, heart rate, total bilirubin, lactate, creatinine, white blood cell count, international normalized ratio (INR), vasopressor use, urine output, and the Glasgow Coma Scale (GCS) score. The model achieved an AUROC of 0.822 (95% confidence interval [CI]: 0.797–0.847) in the training cohort and 0.810 (95% CI: 0.777–0.843) in the temporal validation cohort. External validation yielded AUROCs of 0.777 (95% CI: 0.734–0.821) in the eICU-CRD cohort and 0.763 (95% CI: 0.680–0.846) in the SZPH-ICU cohort. The proposed model demonstrated superior discriminative performance compared with existing prognostic scores.

**Conclusions:**

This validated multivariable prediction model accurately estimates in-hospital mortality in ICU patients with cirrhosis and sepsis, supporting early risk stratification and more efficient allocation of clinical resources.

## Introduction

Cirrhosis, the terminal stage of multiple chronic liver diseases, represents a major global public health burden owing to its high morbidity and mortality (1–3). Patients with cirrhosis are particularly susceptible to infections and subsequent sepsis as a result of intrinsic immune dysfunction, impaired intestinal barrier integrity, and systemic inflammation (4, 5). Sepsis is not only a frequent and severe complication in this patient population but also a leading cause of admission to the intensive care unit (ICU) (6–8). The concurrence of cirrhosis and sepsis initiates a vicious cycle that exacerbates multi-organ failure and significantly increases mortality risk (9–11). Notably, septic shock in patients with cirrhosis is associated with in-hospital mortality rates of up to 70% (12). Accordingly, early and accurate identification of high-risk patients is crucial for improving clinical outcomes and optimizing healthcare resource utilization.

In clinical practice, several scoring systems are used to estimate prognosis in critically ill patients. These systems encompass general ICU scores such as the Sequential Organ Failure Assessment (SOFA) and Simplified Acute Physiology Score II, as well as liver-specific models such as the Model for End-Stage Liver Disease (MELD) and Child-Pugh score (13–16). However, general scoring systems often fail to capture the pathophysiological complexity of cirrhosis, whereas liver-specific scores may not comprehensively reflect sepsis-induced systemic organ dysfunction. Consequently, their prognostic performance in cirrhotic patients with sepsis remains suboptimal. For example, SOFA and MELD-Na scores have demonstrated area under the receiver operating characteristic curve (AUROC) values of only 0.684 and 0.672, respectively (17). Recently, efforts have been made to develop more tailored predictive tools. Lin et al. developed a nomogram based on the MIMIC-IV database that achieved an AUROC of 0.827, outperforming SOFA and MELD-Na (17). Similarly, the LIVERAID-ICU score, proposed by Hoppmann et al. (18) integrates the Child-Pugh score, serum urea, and respiratory parameters to predict outcomes in cirrhotic patients with infections in the ICU, demonstrating an AUROC of 0.830 and superior performance compared with SOFA and MELD. Despite these advances, many existing models are often limited by single-center data, modest sample sizes, and insufficient external validation, which restricts their generalizability.

Therefore, this study aimed to develop and validate a novel, clinically practical, multivariable prediction model for in-hospital mortality among ICU patients with cirrhosis and sepsis. By incorporating readily available and objective parameters, we seek to provide a robust and reliable tool for early risk stratification and informed clinical decision-making in this high-risk population.

## Materials and methods

### Study design and data sources

This multicentre retrospective cohort study integrated data from three ICU databases: the Medical Information Mart for Intensive Care IV (MIMIC-IV v3.1), the eICU Collaborative Research Database (eICU-CRD v2.0), and the Shenzhen People's Hospital ICU (SZPH-ICU; a tertiary academic medical centre in China). The MIMIC-IV database contains data on over 90,000 ICU admissions at Beth Israel Deaconess Medical Centre, Boston, Massachusetts, USA, between 2008 and 2022 (19). The eICU-CRD is a multicentre database comprising over 200,000 ICU admissions from 208 hospitals across the United States between 2014 and 2015 (20). The SZPH-ICU database includes over 8,000 ICU admissions recorded between 2014 and 2023. All databases provided demographic characteristics, vital signs, treatment measures, nursing records, and laboratory data. The MIMIC-IV database was fully anonymized, with no personally identifiable information retained, and ethical approval was obtained from the Institutional Review Boards (IRBs) of Beth Israel Deaconess Medical Centre and the Massachusetts Institute of Technology. The eICU-CRD is a publicly available, de-identified database (HIPAA certification number 1031219-2) and was exempt from IRB review. The use of the SZPH-ICU dataset was approved by the local ethics committee (approval number: LL-KY-2025288-01), and the requirement for informed consent was waived because of the retrospective study design. This study strictly adhered to the Transparent Reporting of a Multivariable Prediction Model for Individual Prognosis or Diagnosis guidelines (21).

### Study population

Patients with cirrhosis who met the Sepsis-3 criteria on the first day of ICU admission were included. Cirrhosis was identified using International Classification of Diseases (ICD-9 or ICD-10) codes from patient discharge records (Supplementary Table 1). Patients aged < 18 years or with an ICU stay of < 24 h were excluded. For patients with multiple ICU admissions, only data from the first admission were included.

### Primary outcome

The primary outcome was all-cause in-hospital mortality. Out-of-hospital follow-up data were unavailable in both the eICU-CRD and SZPH-ICU databases, and only 28-day mortality was available in the MIMIC-IV database.

### Variable selection and data extraction

Based on prior literature and data availability, 25 candidate variables were prespecified (13–18). These included demographic characteristics (age, sex, and body mass index); vital signs (mean arterial pressure, respiratory rate, body temperature, oxygen saturation, and heart rate); laboratory parameters [total bilirubin, albumin, lactate, creatinine, white blood cell count, sodium, and international normalized ratio (INR)]; clinical interventions (renal replacement therapy, vasopressor use, and invasive mechanical ventilation); emergency admission status; and cirrhosis-related complications (ascites, hepatic encephalopathy, viral hepatitis, and alcohol abuse). The Glasgow Coma Scale (GCS) score and urine output were also included. All variables were extracted from the first 24 h following ICU admission. Vital signs were averaged over this period, and laboratory values were recorded as the most extreme values reflecting disease severity (e.g., maximum or minimum values).

### Data preprocessing and missing data imputation

For continuous variables (e.g., laboratory measurements and vital signs), outliers were defined as values below the first percentile or above the 99th percentile and were winsorized to the corresponding percentile thresholds. Missing values were imputed using multiple imputation via the “missForest” package in R, which implements a random forest-based algorithm (22).

### Model development and validation

The MIMIC-IV cohort was chronologically divided into a training set (2008–2016) and a temporal validation set (2017–2022). External validation was performed using the eICU-CRD and SZPH-ICU cohorts. Variable selection was conducted exclusively in the MIMIC-IV training set. To simplify the model and enhance its applicability, least absolute shrinkage and selection operator (LASSO) regression with 10-fold cross-validation was applied to identify the most predictive variables. A multivariable logistic regression model was then constructed using the LASSO-selected variables and subsequently transformed into a nomogram for clinical use. Model performance was evaluated using AUROC values, the Brier score, calibration curves, and decision curve analysis. To convert the model's predicted probabilities into binary outcomes (high vs. low risk), the optimal probability threshold was determined using the Youden index. The Youden index (*J* = sensitivity + specificity – 1) identifies the point on the ROC curve that maximizes the combined sensitivity and specificity, thereby providing an optimal balance (23). A web-based risk calculator was developed to facilitate bedside application.

### Comparison with existing scoring systems

The discriminatory performance of the proposed model was compared, using AUROC values, with the following established scoring systems: MELD, MELD-Na, SOFA, Albumin-Bilirubin (ALBI) score, Child-Pugh score, Chronic Liver Failure–Organ Failure (CLIF-OF) score, CLIF Consortium Acute-on-Chronic Liver Failure (CLIF-C ACLF) score, and CLIF-SOFA score (24–26). DeLong tests were used to compare the AUROC values between the different scoring systems. To further quantify the improvement in risk stratification capability of the new model compared to the traditional scoring systems, net reclassification improvement (NRI) was calculated (27).

### Statistical analysis

Categorical variables are presented as frequencies (percentages), and continuous variables as medians (interquartile ranges). Group comparisons were conducted using the chi-square test for categorical variables and the *t*-test or Mann–Whitney *U*-test for continuous variables, as appropriate. All analyses were conducted using R software (version 4.4.0). A two-sided *p*-value < 0.05 was considered statistically significant.

## Results

### Non-survivors presented with worse clinical profiles and higher severity scores

**Figure 1:**
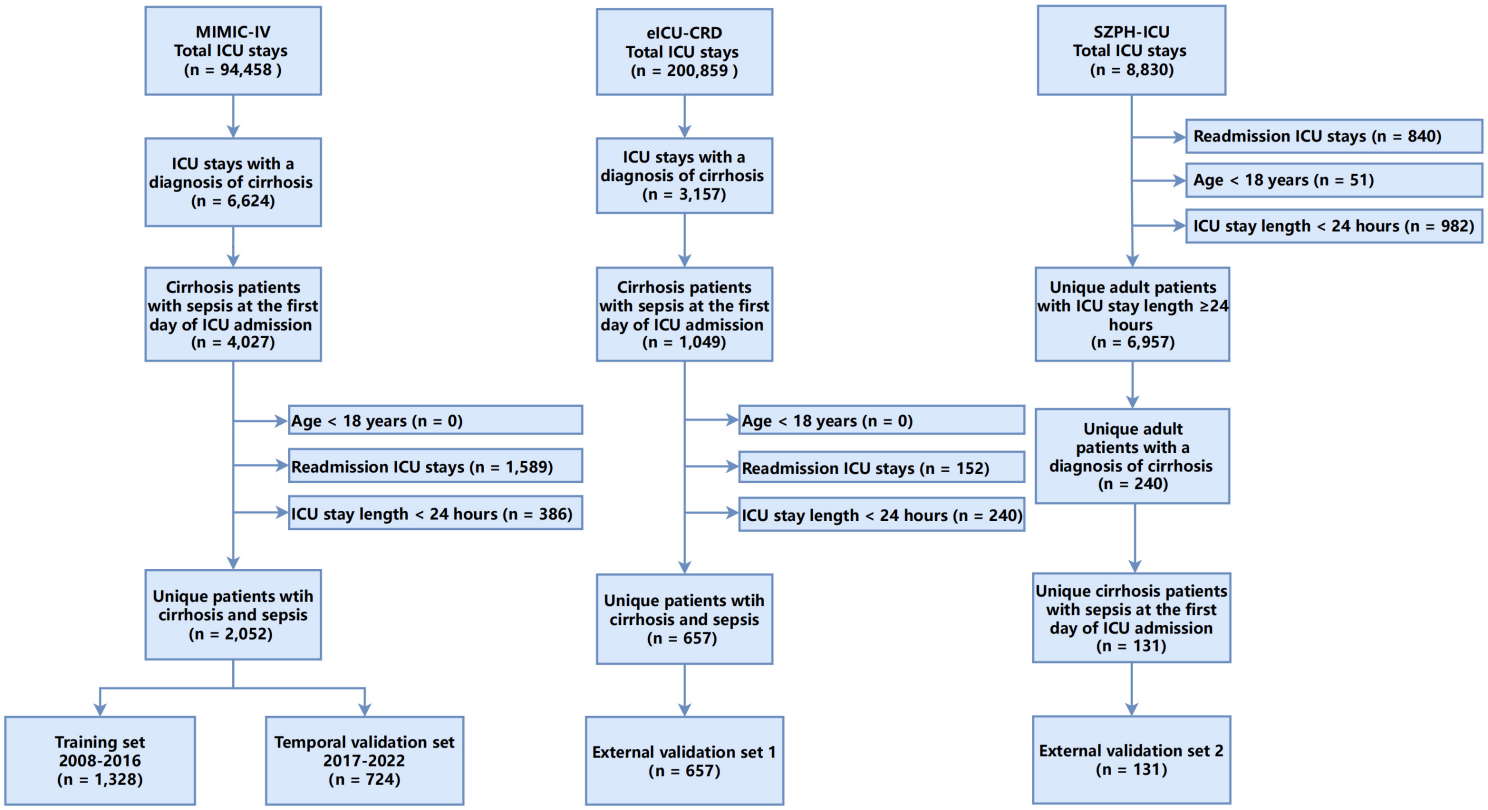
Flowchart of the study.

A total of 2,052 adult ICU patients with cirrhosis or sepsis from the MIMIC-IV database were included in the analysis (Figure 1). The cohort was divided into a training set (2008–2016; *n* = 1,328) and a temporal validation set (2017–2022; *n* = 724). The overall median age was 60.0 years [interquartile range (IQR): 53.0–67.0 years], and 65.1% of patients were men. In the training set, 319 deaths were recorded, corresponding to an in-hospital mortality rate of 24.0%, whereas the temporal validation set comprised 260 deaths (mortality rate: 35.9%).

As Table 1 shows, patients who died during hospitalization exhibited significantly worse clinical indicators and higher disease severity scores than survivors in the training set. Non-survivors demonstrated greater haemodynamic instability, reflected by lower mean arterial pressure and higher heart rate. Respiratory impairment was also more pronounced among non-survivors, with elevated respiratory rates and reduced peripheral oxygen saturation. Laboratory indices indicated more severe hepatic and renal dysfunction as well as heightened systemic inflammation among non-survivors, including significantly higher levels of total bilirubin, lactate, creatinine, white blood cell counts, and INR, along with a higher proportion of patients with 24 h urine output < 400 ml. Therapeutic interventions, such as renal replacement therapy, vasopressor use, and invasive mechanical ventilation, were significantly more frequent in the non-survivor group. Similar patterns were observed in the temporal validation set (Table 1). In the eICU-CRD external validation cohort (*n* = 657), 170 deaths occurred (mortality rate: 25.9%); the median age was 59 years, and 61.6% of patients were men. The SZPH-ICU validation cohort (*n* = 131; 74.8% men) had a median age of 61 years and included 50 deaths (mortality rate: 38.2%). In both external validation cohorts, non-survivors consistently exhibited more severe organ dysfunction and higher disease severity scores (Supplementary Tables 2, 3). These findings are consistent with clinical expectations, whereby greater physiological derangement and more advanced organ dysfunction are typically associated with an increased risk of mortality.

**Table 1:** Patient characteristics in the training and temporal validation cohorts.

**Variable**	**Training set**				**Temporal validation set**			
	**Overall (*****n*** = **1,328)**	**Survival (*****n*** = **1,009)**	**Death (*****n*** = **319)**	***p*** **-value**	**Overall (*****n*** = **724)**	**Survival (*****n*** = **464)**	**Death (*****n*** = **260)**	***p*** **-value**
Age, years	60.0 [53.0, 67.0]	60.0 [53.0, 67.0]	60.0 [53.0, 70.0]	0.171	58.0 [49.8, 65.0]	58.0 [50.0, 65.0]	59.5 [49.0, 66.0]	0.288
Sex, male	865 (65.1)	655 (64.9)	210 (65.8)	0.817	468 (64.6)	316 (68.1)	152 (58.5)	0.012
BMI, kg/m^3^	28.2 [24.4, 33.0]	28.1 [24.4, 32.8]	28.4 [24.8, 33.6]	0.332	28.7 [24.4, 33.7]	28.4 [24.1, 33.0]	30.2 [24.7, 35.7]	0.005
Mean arterial pressure, mmHg	73.0 [68.0, 81.0]	74.0 [68.0, 82.0]	71.0 [66.0, 77.0]	< 0.001	74.0 [70.0, 81.0]	75.0 [71.0, 82.0]	72.0 [67.0, 77.0]	< 0.001
Heart rate, beats/min	87.4 [75.9, 99.2]	85.5 [75.2, 97.0]	94.9 [80.5, 104.5]	< 0.001	89.7 [78.9, 101.3]	86.8 [77.5, 99.0]	92.7 [83.4, 104.8]	< 0.001
Respiratory rate, breaths/min	18.0 [16.0, 21.0]	18.0 [16.0, 21.0]	20.0 [17.0, 24.0]	< 0.001	19.0 [16.0, 22.0]	18.0 [16.0, 21.0]	20.0 [17.0, 23.2]	< 0.001
Body temperature, °C	36.8 [36.5, 37.1]	36.8 [36.6, 37.1]	36.7 [36.4, 36.9]	< 0.001	36.8 [36.6, 37.1]	36.9 [36.7, 37.1]	36.7 [36.5, 36.9]	< 0.001
SpO_2_, %	97.0 [96.0, 99.0]	98.0 [96.0, 99.0]	97.0 [95.0, 98.0]	< 0.001	97.0 [95.0, 99.0]	97.0 [96.0, 99.0]	96.0 [95.0, 98.0]	< 0.001
Urine output < 400 ml	205 (15.4)	101 (10.0)	104 (32.6)	< 0.001	186 (25.7)	77 (16.6)	109 (41.9)	< 0.001
GCS score	15.0 [14.0, 15.0]	15.0 [14.0, 15.0]	15.0 [13.0, 15.0]	0.001	15.0 [13.0, 15.0]	15.0 [14.0, 15.0]	14.0 [12.0, 15.0]	< 0.001
Total bilirubin, μmol/L	54.7 [27.4, 116.3]	47.9 [23.9, 92.3]	99.2 [39.3, 208.6]	< 0.001	80.4 [34.2, 199.7]	68.6 [27.4, 132.1]	156.5 [56.4, 342.4]	< 0.001
Albumin, g/L	28.0 [25.0, 31.6]	28.3 [25.0, 32.0]	27.0 [23.0, 30.3]	< 0.001	28.0 [24.0, 31.9]	28.0 [24.0, 31.0]	27.8 [23.0, 32.8]	0.853
Lactate, mmol/L	3.3 [2.3, 4.6]	3.2 [2.3, 4.2]	4.0 [2.7, 6.2]	< 0.001	4.0 [2.9, 5.6]	3.8 [2.9, 5.2]	4.6 [3.3, 6.4]	< 0.001
Creatinine, μmol/L	114.9 [79.6, 212.2]	106.1 [70.7, 176.8]	176.8 [106.1, 291.7]	< 0.001	150.3 [97.2, 256.4]	132.6 [79.6, 214.4]	194.5 [130.4, 329.3]	< 0.001
WBC, × 10^9^/L	11.4 [7.7, 17.1]	10.9 [7.5, 16.2]	13.0 [9.1, 20.3]	< 0.001	14.8 [9.9, 20.9]	14.1 [9.2, 19.6]	16.4 [12.1, 23.3]	< 0.001
Sodium, mmol/L	136.0 [131.0, 139.0]	136.0 [132.0, 139.0]	134.0 [129.0, 138.0]	< 0.001	135.0 [130.0, 139.0]	135.0 [130.0, 138.0]	134.0 [130.0, 140.0]	0.825
International normalized ratio	1.8 [1.4, 2.3]	1.7 [1.4, 2.1]	2.1 [1.7, 3.0]	< 0.001	2.1 [1.6, 2.7]	1.9 [1.5, 2.5]	2.4 [2.0, 3.3]	< 0.001
RRT	120 (9.0)	78 (7.7)	42 (13.2)	0.005	131 (18.1)	54 (11.6)	77 (29.6)	< 0.001
Vasopressor	629 (47.4)	405 (40.1)	224 (70.2)	< 0.001	344 (47.5)	177 (38.1)	167 (64.2)	< 0.001
IMV	1,101 (82.9)	818 (81.1)	283 (88.7)	0.002	584 (80.7)	360 (77.6)	224 (86.2)	0.007
Presence of ascites	615 (46.3)	434 (43.0)	181 (56.7)	< 0.001	449 (62.0)	273 (58.8)	176 (67.7)	0.023
Hepatic encephalopathy	236 (17.8)	172 (17.0)	64 (20.1)	0.252	105 (14.5)	70 (15.1)	35 (13.5)	0.627
Viral hepatitis history	475 (35.8)	369 (36.6)	106 (33.2)	0.308	118 (16.3)	87 (18.8)	31 (11.9)	0.023
Alcohol abuse history	750 (56.5)	557 (55.2)	193 (60.5)	0.11	497 (68.6)	313 (67.5)	184 (70.8)	0.402
Emergency admission	1,158 (87.2)	875 (86.7)	283 (88.7)	0.405	547 (75.6)	332 (71.6)	215 (82.7)	0.001
MELD	21.0 [15.0, 29.0]	20.0 [14.0, 26.0]	29.0 [22.0, 35.0]	< 0.001	26.0 [19.0, 35.0]	23.0 [17.0, 30.0]	33.5 [25.0, 40.0]	< 0.001
MELD-Na	23.7 [16.6, 30.8]	21.1 [15.0, 28.0]	30.9 [24.1, 36.2]	< 0.001	28.5 [21.0, 35.6]	25.3 [18.7, 32.0]	34.0 [27.4, 40.0]	< 0.001
SOFA	8.0 [6.0, 11.0]	8.0 [5.0, 10.0]	11.0 [8.0, 14.0]	< 0.001	10.0 [7.0, 13.0]	9.0 [6.0, 11.0]	12.0 [9.0, 15.0]	< 0.001
Albumin-bilirubin	−1.0 [−1.6, −0.3]	−1.1 [−1.7, −0.5]	−0.7 [−1.3, 0.0]	< 0.001	−0.9 [−1.4, −0.3]	−1.0 [−1.5, −0.5]	−0.8 [−1.4, −0.1]	0.001
Child-Pugh	9.0 [7.0, 11.0]	8.0 [7.0, 10.0]	10.0 [8.0, 11.0]	< 0.001	10.0 [8.0, 11.0]	9.0 [7.0, 11.0]	10.0 [9.0, 11.0]	< 0.001
CLIF-OF	10.0 [9.0, 12.0]	10.0 [8.0, 11.0]	12.0 [10.0, 14.0]	< 0.001	11.0 [9.0, 13.0]	10.0 [9.0, 12.0]	13.0 [11.0, 14.0]	< 0.001
CLIF-C ACLF	54.0 [47.0, 62.0]	52.0 [45.0, 58.0]	62.0 [55.0, 67.0]	< 0.001	56.0 [49.0, 63.0]	53.0 [46.0, 59.0]	63.0 [56.0, 68.0]	< 0.001
CLIF-SOFA	10.0 [7.0, 13.0]	9.0 [7.0, 12.0]	13.0 [10.0, 15.0]	< 0.001	10.0 [8.0, 14.0]	9.0 [7.0, 12.0]	13.0 [10.0, 15.0]	< 0.001

Values are detailed as *N* (%) or median [IQR].

### Variable selection via LASSO regression identified 13 predictors for model construction

**Figure 2:**
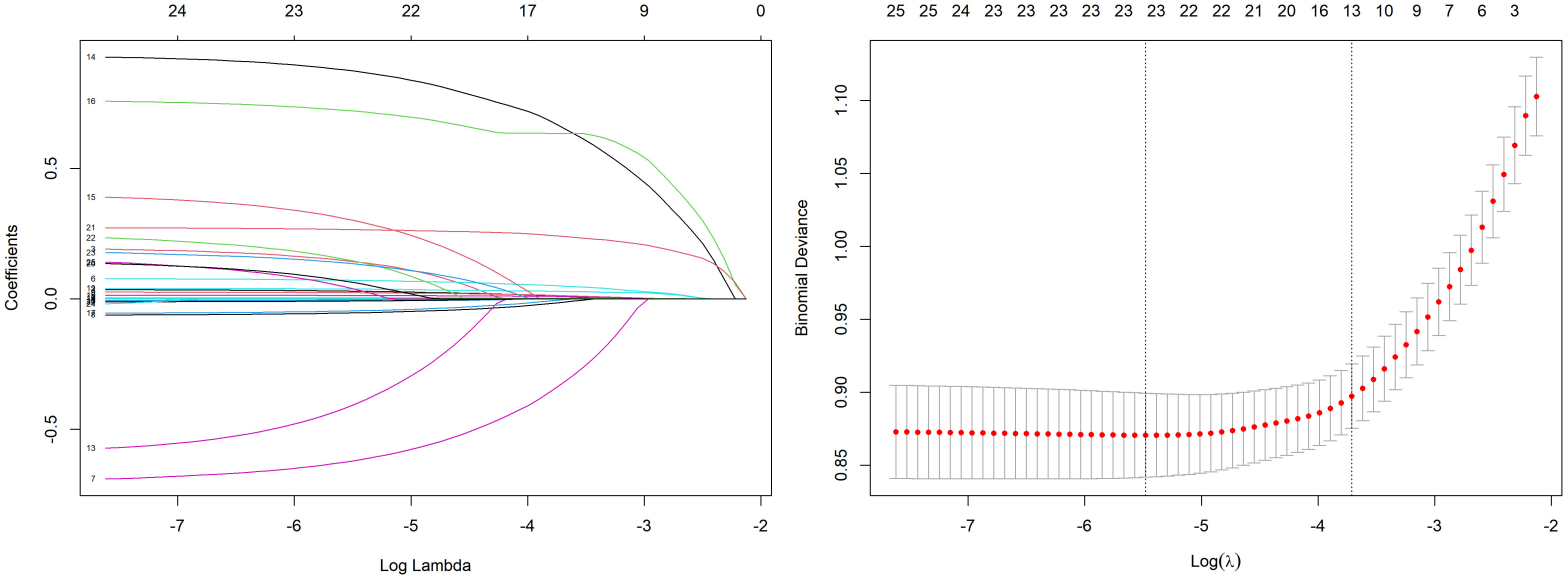
Variable selection using the LASSO regression with 10-fold cross-validation.

Variable selection was performed using LASSO regression in the training set to identify predictors most strongly associated with in-hospital mortality (Figure 2). The optimal regularization parameter (λ_0.1se_ = 0.024) was determined via 10-fold cross-validation, yielding 13 variables with non-zero coefficients: age, respiratory rate, body temperature, oxygen saturation, heart rate, total bilirubin, lactate, creatinine, white blood cell count, INR, vasopressor use, urine output, and GCS score.

### A multivariable prediction model was constructed and visualized as a nomogram

**Figure 3:**
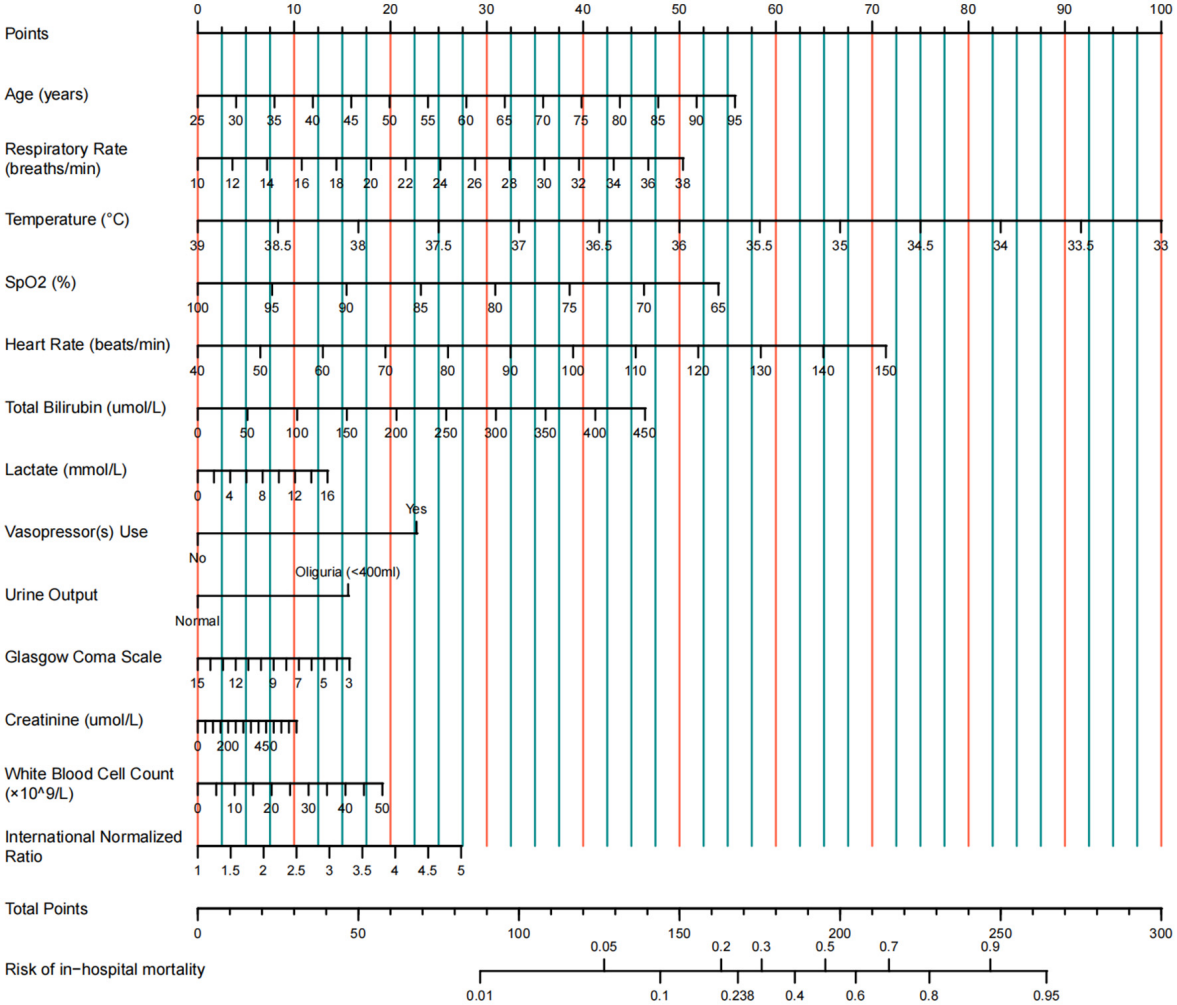
Nomogram for predicting in-hospital mortality in ICU patients with cirrhosis and sepsis.

A multivariable logistic regression model was constructed using the 13 predictors identified by LASSO (coefficients presented in Table 2). For clinical applicability, the model was visualized as a nomogram (Figure 3). This nomogram serves as a graphical computational tool, enabling clinicians to assign scores to each variable (e.g., age, vital signs, laboratory values) according to an individual patient's characteristics. The scores are then summed to obtain a total score, which corresponds to the predicted probability of mortality on the total score axis. This approach visually quantifies the relative contribution of each predictor to mortality risk and facilitates rapid, individualized risk assessment.

**Table 2:** Multivariable logistic regression analysis of factors associated with in-hospital mortality in cirrhotic patients with sepsis, developed from LASSO-selected variables.

**Variable**	**Regression coefficient**	**Odds ratio (95% CI)**	***p*-value**
Intercept	23.858	–	–
Age	0.034	1.035 (1.021–1.049)	< 0.001
Respiratory rate	0.077	1.080 (1.041–1.121)	< 0.001
Body temperature	−0.713	0.490 (0.362–0.661)	< 0.001
SpO_2_	−0.066	0.936 (0.877–0.997)	0.041
Heart rate	0.028	1.028 (1.018–1.039)	< 0.001
Total bilirubin	0.004	1.004 (1.003–1.006)	< 0.001
Lactate	0.036	1.037 (0.976–1.101)	0.241
Vasopressor	0.971	2.640 (1.922–3.645)	< 0.001
Urine output < 400 ml	0.667	1.949 (1.284–2.959)	0.002
GCS score	−0.056	0.945 (0.899–0.996)	0.031
Creatinine	0.001	1.001 (0.999–1.002)	0.262
WBC	0.016	1.017 (0.998–1.036)	0.085
International normalized ratio	0.292	1.339 (1.118–1.604)	0.001

### The nomogram showed robust discriminative ability and calibration across all cohorts

**Figure 4:**
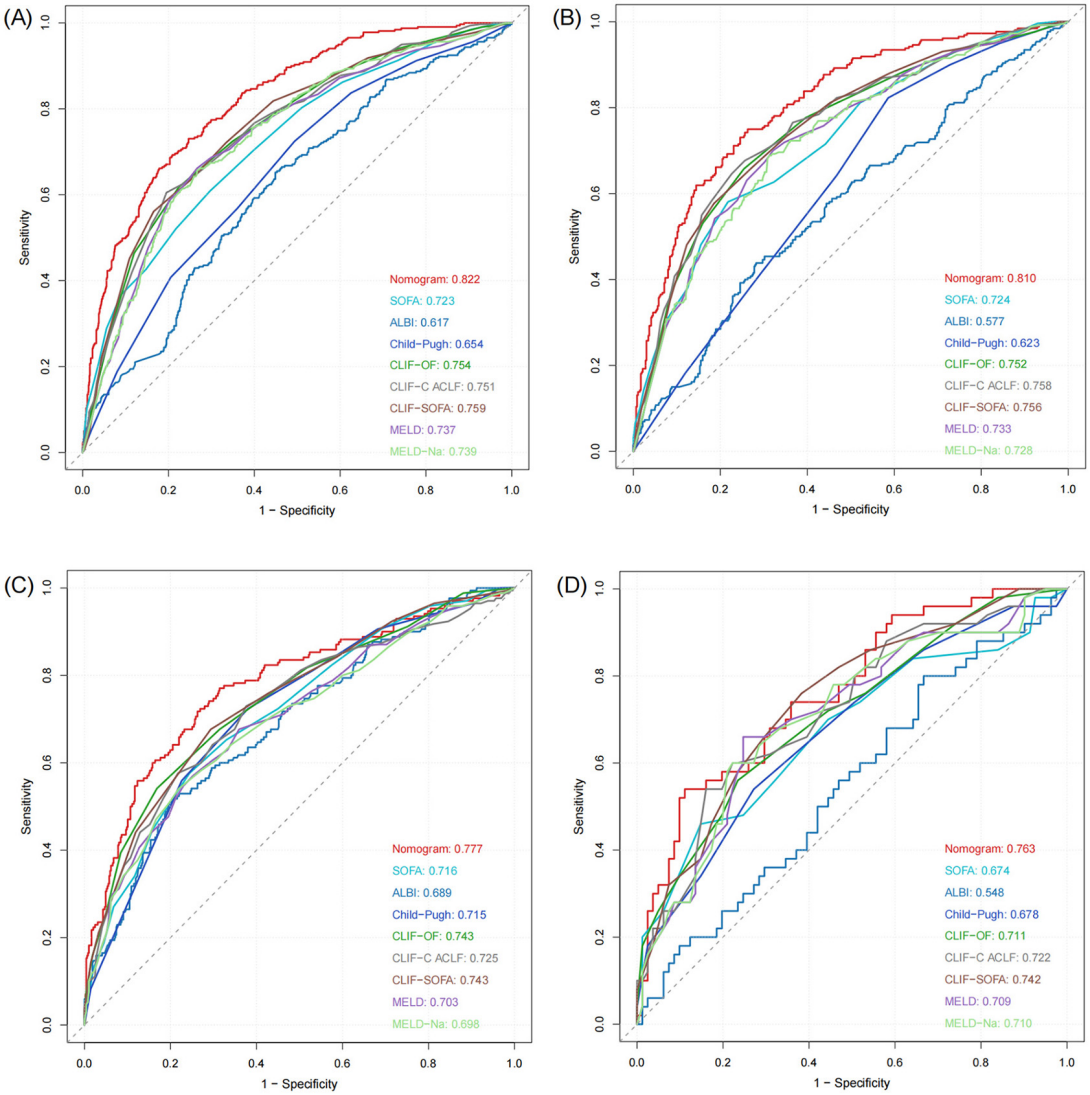
ROC curves of the model in the training and validation sets. **(A)** Training set. **(B)** Temporal validation set. **(C)** External validation set (eICU-CRD). **(D)** External validation set (SZPH-ICU).

**Figure 5:**
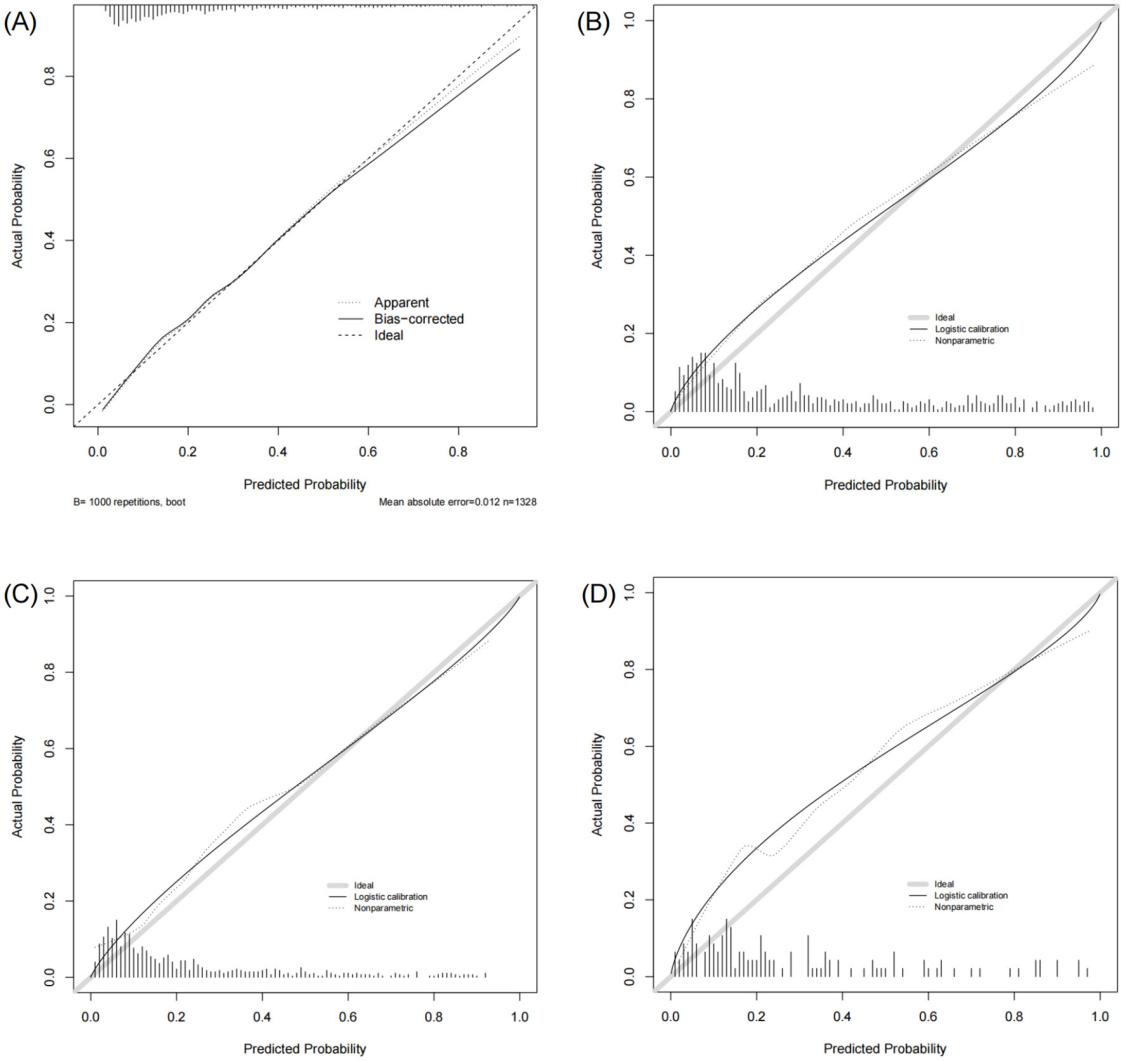
Calibration curves of the model in the training and validation sets. **(A)** Training set. **(B)** Temporal validation set. **(C)** External validation set (eICU-CRD). **(D)** External validation set (SZPH-ICU).

The nomogram demonstrated strong predictive performance in both the training and validation cohorts (Table 3 and Figure 4). In the training set, an AUROC of 0.822 (95% CI: 0.797–0.847) and a Brier score of 0.135 indicated excellent discrimination and calibration. Despite differences in baseline characteristics between the temporal validation set and the training set (Table 1), performance remained stable in the temporal validation cohort (AUROC = 0.810, 95% CI: 0.777–0.843; Brier score = 0.169). External validation in the eICU-CRD cohort yielded an AUROC of 0.777 (95% CI: 0.734–0.821; Brier score = 0.150), while validation in the SZPH-ICU cohort yielded an AUROC of 0.763 (95% CI: 0.680–0.846; Brier score = 0.200). Figure 5 presents the calibration curves for all datasets, illustrating the concordance between the model's predicted probabilities and the actual observed mortality rates. Across all datasets, the calibration curves closely aligned with the diagonal line, signifying a high degree of calibration in the model's predictions. In the training dataset, the Youden index was computed across a range of probability thresholds. The maximum value (0.48), corresponding to a sensitivity of 73% and a specificity of 75%, was observed at a threshold of 0.238. This value was therefore selected as the optimal cut-off for distinguishing high-risk and low-risk patients.

**Table 3:** Performance of the nomogram in predicting in-hospital mortality in the training and validation sets.

**Datasets**	**AUROC (95% CI)**	**Brier score**
Training set	0.822 (0.797–0.847)	0.135
Temporal validation set	0.810 (0.777–0.843)	0.169
eICU-CRD validation set	0.777 (0.734–0.821)	0.150
SZPH-ICU validation set	0.763 (0.680–0.846)	0.200

### The nomogram showed superior predictive accuracy compared to existing clinical scores

The nomogram was systematically compared with eight established scoring systems: SOFA, ALBI, Child–Pugh, CLIF-OF, CLIF-C ACLF, CLIF-SOFA, MELD, and MELD-Na (Figure 4). In the training set, the nomogram (AUROC = 0.822) significantly outperformed all comparator scores (*p* < 0.05), with the best-performing conventional score (CLIF-SOFA) achieving an AUROC of 0.759. Similar superiority was observed in the temporal validation set (AUROC = 0.810; *p* < 0.05 for all comparisons).

In the eICU-CRD cohort, the nomogram achieved an AUROC of 0.777, which was significantly higher than those of SOFA, ALBI, Child–Pugh, CLIF-C ACLF, MELD, and MELD-Na (all *p* < 0.05). Although the differences compared with CLIF-OF (*p* = 0.105) and CLIF-SOFA (*p* = 0.107) were not statistically significant, the nomogram still exhibited numerical superiority. In the SZPH-ICU cohort, the nomogram (AUROC = 0.763) did not differ significantly from SOFA (*p* = 0.062), Child–Pugh (*p* = 0.093), CLIF-OF (*p* = 0.158), CLIF-C ACLF (*p* = 0.194), MELD (*p* = 0.218), or MELD-Na (*p* = 0.223), but significantly outperformed ALBI (*p* < 0.05) and consistently yielded the highest AUROC among all scores.

The incremental value of the nomogram over CLIF-SOFA was further assessed using the NRI index. Table 4 shows that in both the training and temporal validation sets, the NRI was significantly greater than zero (training set: NRI = 0.097, *p* = 0.002; temporal validation set: NRI = 0.092, *p* = 0.011), indicating that approximately 9–10% of patients were more accurately reclassified by the nomogram. In the external validation cohorts, the eICU-CRD cohort dataset showed a positive but non-significant NRI (NRI = 0.053, *p* = 0.234), whereas the SZPH-ICU cohort (*n* = 131) demonstrated no reclassification benefit (NRI = −0.001, *p* = 0.994). Overall, the NRI findings were consistent with the AUROC comparisons, supporting the nomogram's superior risk reclassification performance over CLIF-SOFA in the primary cohorts.

**Table 4:** The NRI of the developed model compared with the CLIF-SOFA score.

**Datasets**	**Sample size**	**NRI**	***p*-value**
Training set	1,328	0.097	0.002
Temporal validation set	724	0.092	0.011
eICU-CRD validation set	657	0.053	0.234
SZPH-ICU validation set	131	−0.001	0.994

### Decision curve analysis confirmed the clinical net benefit of the model over default strategies

**Figure 6:**
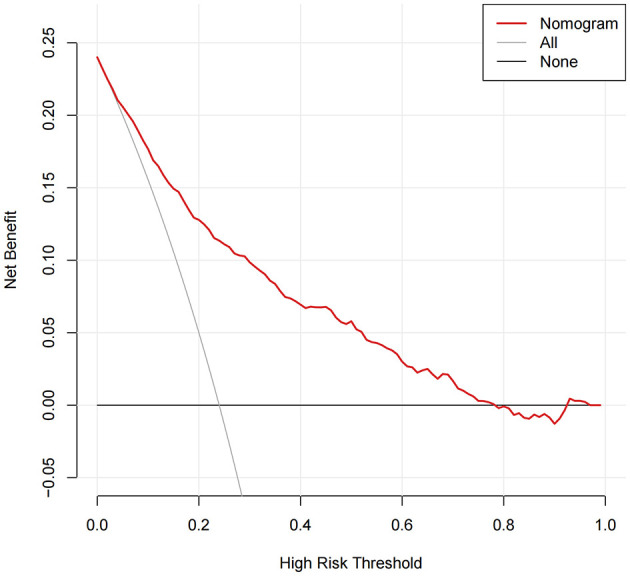
Clinical utility of the model as assessed using decision curve analysis across a range of threshold probabilities.

Decision curve analysis was used to evaluate the clinical net benefit of the model across a range of decision thresholds. As Figure 6 illustrates, over a broad range of threshold probabilities (approximately 0.05–0.78), employing this model for risk stratification—by intervening in patients whose predicted probabilities exceed the selected threshold—yielded a higher net clinical benefit than either treating all patients or treating none. At threshold probabilities above 0.78, the model's net benefit diminished, aligning closely with the “treat-none” strategy. This outcome is anticipated, as such high thresholds are rarely adopted in routine clinical practice when initiating therapy. Overall, these findings underscore the clinical utility of applying the proposed model.

### The model was implemented as an accessible web-based tool for clinical decision support

**Figure 7:**
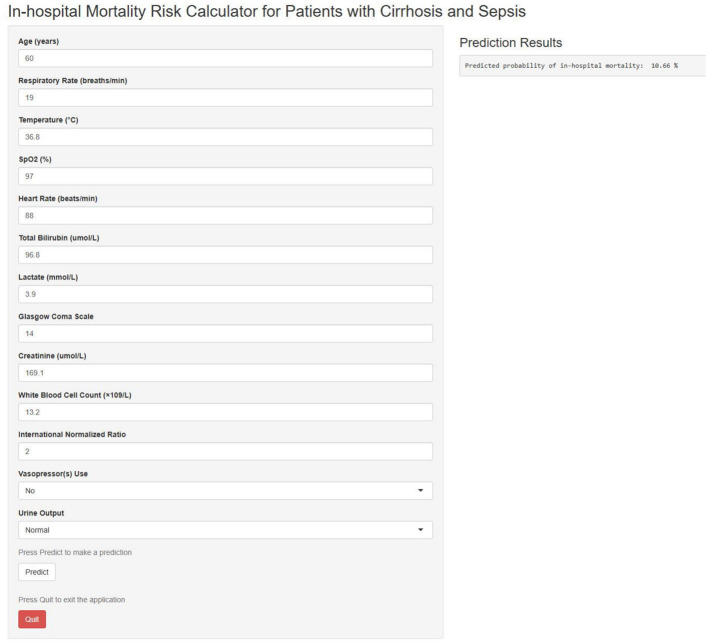
Web-based in-hospital mortality prediction calculator.

To facilitate clinical translation, an interactive web-based calculator was developed using the nomogram and is accessible at https://szph-icu.shinyapps.io/Cirrhosis-Sepsis-ICU/ (Figure 7). This tool enables clinicians to input patient-specific parameters, including age, vital signs, and laboratory parameters, to generate real-time estimates of in-hospital mortality risk. Consequently, it aids bedside decision-making and facilitates individualized treatment planning.

## Discussion

In this multicentre retrospective study, we successfully developed and validated a novel multivariable logistic regression model to predict in-hospital mortality in ICU patients with cirrhosis and sepsis. The model incorporated 13 routinely available clinical variables and demonstrated excellent and stable predictive performance in both internal and external validation cohorts (eICU-CRD and SZPH-ICU), with AUROCs ranging from 0.763 to 0.822. This performance is comparable with the AUROC (0.830) reported for a recently published predictive model in ICU patients with cirrhosis and infection (18). Compared with eight existing general or liver-specific prognostic scoring systems (SOFA, ALBI, Child–Pugh, CLIF-OF, CLIF-C ACLF, CLIF-SOFA, MELD, and MELD-Na), our model exhibited significantly superior or at least comparable discriminative ability. This advantage was not only evident in AUROC comparisons but was further supported by the NRI metric, indicating enhanced risk stratification. Furthermore, decision curve analysis demonstrated a meaningful net clinical benefit across a wide range of threshold probabilities. Application of the model for risk stratification may facilitate more individualized clinical management. High-risk patients may benefit from earlier and more intensive monitoring and interventions, including enhanced haemodynamic surveillance, timely organ support, and optimisation of antimicrobial therapy. Conversely, identification of low-risk patients may help avoid unnecessary overtreatment, thereby improving resource utilization and enabling prioritization of patients with the greatest clinical need (28).

Patients with cirrhosis and sepsis typically have a poor prognosis, with mortality largely driven by the combined effects of hepatic dysfunction and sepsis-induced multiorgan failure (7, 29–31). Consistent with previous research, the predictors identified by our model—including GCS score, renal function (creatinine), hyperbilirubinaemia, coagulation status (INR), requirement for circulatory support (vasopressor use), and indicators of inflammation and metabolism (lactate)—are well-established determinants of outcome in critically ill patients with cirrhosis (32–34). These findings reinforce the central role of multi-organ dysfunction, particularly involving the liver, kidneys, cardiovascular system, and central nervous system, in shaping prognosis in this population. The novelty of our model lies in its comprehensive and generalizable design, integrating indicators of hepatic dysfunction (e.g., total bilirubin and INR) with key physiological parameters reflecting systemic inflammatory responses, such as respiratory rate, temperature, and heart rate. This integrative approach addresses the limitations of traditional liver-based scoring systems in capturing the systemic manifestations of sepsis and may explain the superior performance of our model compared with scores focused primarily on organ failure (e.g., SOFA) or hepatic function alone (e.g., MELD). In summary, our model highlights the pivotal importance of multisystem parameters, including neurological, hepatic, renal, and cardiovascular functions, in enhancing the accuracy of outcome predictions for patients with cirrhosis and sepsis.

Notably, body temperature emerged as one of the strongest predictors and was inversely associated with in-hospital mortality. Patients with decompensated cirrhosis often exhibit an attenuated febrile response due to immune dysfunction (4). Previous studies have shown that hypothermia is associated with increased mortality in sepsis, potentially reflecting profound immune suppression (35, 36). Hypothermic septic phenotypes have been linked to reduced levels of both pro-inflammatory and anti-inflammatory cytokines, suggesting a globally suppressed immune response. In cirrhotic patients with pre-existing immunodeficiency, hypothermia may be even more detrimental (37). Another key variable identified was heart rate. Tachycardia is a fundamental criterion of the systemic inflammatory response syndrome and also reflects the hyperdynamic circulatory state associated with portal hypertension in cirrhosis (38, 39). In cirrhotic patients with sepsis, an elevated heart rate captures both underlying haemodynamic alterations and the magnitude of the systemic stress response to infection, making it a clinically relevant indicator of disease severity and prognosis (40, 41). Age also emerged as an independent adverse prognostic factor. Aging is associated with immunosenescence, characterized by declines in both innate and adaptive immunity, increased susceptibility to infections, and reduced physiological reserve (42). Older patients with cirrhosis frequently have multiple comorbidities and limited capacity to tolerate septic insults, predisposing them to organ failure and poorer clinical outcomes (43).

Previous research has shown that commonly used prognostic models, such as the SOFA and MELD, often demonstrate limited specificity and accuracy when applied to specific subpopulations of cirrhotic patients with sepsis (17). Although several novel scoring systems, including the LIVERAID-ICU score, have been proposed, the present study advances this field in several important respects (17, 18). First, our model was developed using a larger sample size and more diverse data sources, including the MIMIC-IV, eICU-CRD, and SZPH-ICU databases, thereby enhancing robustness and generalizability. Second, we conducted a comprehensive temporal, spatial, and external validation, demonstrating strong generalizability across various time periods and healthcare settings—a crucial step toward clinical implementation that is frequently lacking in previous studies. Notably, the model exhibited stable discriminative performance across internal validation sets from different time frames and across external validation sets from diverse healthcare systems, indicating its ability to capture the core pathophysiological characteristics common to this patient population. Although a modest reduction in the AUROC was observed in the single-center SZPH-ICU external validation cohort (0.763), model performance remained comparable to or superior to that of the leading traditional scoring systems, highlighting its extensive clinical applicability. Such attenuation in external validation is common and may reflect differences in patient characteristics, clinical practice patterns, or data collection procedures. Furthermore, the development of a web-based calculator markedly enhances usability and accessibility, thereby promoting its integration into bedside clinical decision-making processes.

Several limitations should be acknowledged. First, the study relied on retrospectively collected electronic health record data that were not originally designed for this specific research purpose, introducing the potential for information bias due to incomplete recordings or coding errors. Second, although this study utilized multicentre data, the SZPH-ICU cohort originated from a single institution; therefore, further validation in larger, more diverse, and geographically representative populations is warranted. Third, several potentially important prognostic variables, such as the specific etiology of cirrhosis and specific sites of infection, were not consistently available across the included databases. Final, our model, which is based on data from the first 24 h of ICU admission, is static and similar to traditional critical illness scores, allowing early risk identification. However, it doesn't account for treatment responses or disease progression over time. Future research should focus on collecting data at key intervals (e.g., days 7, 14, and 28) and using time-dependent models to create dynamic tools. These tools could offer continuously updated risk assessments, improving the precision and timeliness of clinical decisions.

## Conclusions

We successfully developed and validated a novel multivariable predictive model for in-hospital mortality in ICU patients with cirrhosis and sepsis. This model exhibited robust discrimination, satisfactory calibration, and significant clinical utility, outperforming several existing prognostic scoring systems. By integrating the model into a web-based calculator, we provide clinicians with a user-friendly and bedside-compatible tool for timely risk assessment, enabling early identification of high-risk patients, supporting optimized allocation of treatment resources, and facilitating individualized clinical decision-making. Prospective studies are necessary to further confirm the model's performance and generalizability across diverse clinical settings.

## Data Availability

Publicly available datasets were analyzed in this study. This data can be found here: MIMIC-IV: https://physionet.org/content/mimiciv/3.1/ and eICU-CRD: https://physionet.org/content/eicu-crd/2.0/.

## References

[1] Ginès P Krag A Abraldes JG Solà E Fabrellas N Kamath PS. Liver cirrhosis. Lancet. (2021) 398:1359–76. doi: 10.1016/S0140-6736(21)01374-X 34543610

[2] Asrani SK Devarbhavi H Eaton J Kamath PS. Burden of liver diseases in the world. J Hepatol. (2019) 70:151–71. doi: 10.1016/j.jhep.2018.09.014 30266282

[3] Allen AM Kim WR Moriarty JP Shah ND Larson JJ Kamath PS. Time trends in the health care burden and mortality of acute on chronic liver failure in the United States. Hepatology. (2016) 64:2165–72. doi: 10.1002/hep.28812 27696493

[4] Bajaj JS Kamath PS Reddy KR. The evolving challenge of infections in cirrhosis. N Engl J Med. (2021) 384:2317–30. doi: 10.1056/NEJMra2021808 34133861

[5] Piano S Bunchorntavakul C Marciano S Rajender Reddy K. Infections in cirrhosis. Lancet Gastroenterol Hepatol. (2024) 9:745–57. doi: 10.1016/S2468-1253(24)00078-5 38754453

[6] Choi C Choi DH Spears GM Peeraphatdit TB Serafim LP Gajic O . Relationship between etiology of cirrhosis and survival among patients hospitalized in intensive care units. Mayo Clin Proc. (2022) 97:274–84. doi: 10.1016/j.mayocp.2021.08.025 35090753 PMC8883528

[7] Wong F Bernardi M Balk R Christman B Moreau R Garcia-Tsao G . Sepsis in cirrhosis: report on the 7th meeting of the International Ascites Club. Gut. (2005) 54:718–25. doi: 10.1136/gut.2004.038679 15831923 PMC1774473

[8] Navasa M Fernández J Rodés J. Bacterial infections in liver cirrhosis. Ital J Gastroenterol Hepatol. (1999) 31:616–25. 10604106

[9] Arvaniti V D'Amico G Fede G Manousou P Tsochatzis E Pleguezuelo M . Infections in patients with cirrhosis increase mortality four-fold and should be used in determining prognosis. Gastroenterology. (2010) 139:1246–56.e5. doi: 10.1053/j.gastro.2010.06.019 20558165

[10] Dasgupta A Jones TK Giannini H Bennett R Emre G Ittner CAG . Identifying a unique signature of sepsis in patients with pre-existing cirrhosis. Crit Care. (2025) 29:199. doi: 10.1186/s13054-025-05423-6 40390062 PMC12090594

[11] Gustot T Felleiter P Pickkers P Sakr Y Rello J Velissaris D . Impact of infection on the prognosis of critically ill cirrhotic patients: results from a large worldwide study. Liver Int. (2014) 34:1496–503. doi: 10.1111/liv.12520 24606193

[12] Galbois A Aegerter P Martel-Samb P Housset C Thabut D Offenstadt G . Improved prognosis of septic shock in patients with cirrhosis. Crit Care Med. (2014) 42:1666–75. doi: 10.1097/CCM.0000000000000321 24732239

[13] Vincent J-L Moreno R Takala J Willatts S De Mendonça A Bruining H . The SOFA (sepsis-related organ failure assessment) score to describe organ dysfunction/failure. Intensive Care Med. (1996) 22:707–10. doi: 10.1007/s001340050156 8844239

[14] Le Gall J-R A A new simplified acute physiology score (SAPS II) based on a European/North American multicenter study. JAMA J Am Med Assoc. (1993) 270:2957. doi: 10.1001/jama.1993.03510240069035 8254858

[15] Kamath PS Wiesner RH Malinchoc M Kremers W Therneau TM Kosberg CL . Model to predict survival in patients with end–stage liver disease. Hepatology. (2001) 33:464–70. doi: 10.1053/jhep.2001.22172 11172350

[16] Pugh RNH Murray-Lyon IM Dawson JL Pietroni MC Williams R. Transection of the oesophagus for bleeding oesophageal varices. J Br Surg. (1973) 60:646–9. doi: 10.1002/bjs.1800600817 4541913

[17] Lin H Liao Q Lin X Zhou Y Lin J Xiao X. Development of a nomogram for predicting in-hospital mortality in patients with liver cirrhosis and sepsis. Sci Rep. (2024) 14:9759. doi: 10.1038/s41598-024-60305-1 38684696 PMC11059344

[18] Hoppmann H Zeman F Wittmann D Stöckert P Schlosser-Hupf S Mehrl A . The LIVERAID (LIVER and infectious diseases)-ICU score predicts in-hospital mortality in liver cirrhosis patients with infections in the intensive care unit. BMJ Open Gastroenterol. (2024) 11:e001482. doi: 10.1136/bmjgast-2024-001482 39384247 PMC11481117

[19] Johnson AEW Bulgarelli L Shen L Gayles A Shammout A Horng S . MIMIC-IV, a freely accessible electronic health record dataset. Sci Data. (2023) 10:1. doi: 10.1038/s41597-022-01899-x 36596836 PMC9810617

[20] Pollard TJ Johnson AEW Raffa JD Celi LA Mark RG Badawi O. The eICU collaborative research database, a freely available multi-center database for critical care research. Sci Data. (2018) 5:180178. doi: 10.1038/sdata.2018.178 30204154 PMC6132188

[21] Collins GS Reitsma JB Altman DG Moons KGM. Transparent reporting of a multivariable prediction model for individual prognosis or diagnosis (TRIPOD): the TRIPOD statement. BMJ. (2015) 350:g7594. doi: 10.1136/bmj.g7594 25569120

[22] Stekhoven DJ Bühlmann P. MissForest—non-parametric missing value imputation for mixed-type data. Bioinformatics. (2012) 28:112–8. doi: 10.1093/bioinformatics/btr597 22039212

[23] Staffa SJ Zurakowski D. Statistical development and validation of clinical prediction models. Anesthesiology. (2021) 135:396–405. doi: 10.1097/ALN.0000000000003871 34330146

[24] Johnson PJ Berhane S Kagebayashi C Satomura S Teng M Reeves HL . Assessment of liver function in patients with hepatocellular carcinoma: a new evidence-based approach-the ALBI grade. J Clin Oncol. (2015) 33:550–8. doi: 10.1200/JCO.2014.57.9151 25512453 PMC4322258

[25] Jalan R Saliba F Pavesi M Amoros A Moreau R Ginès P . Development and validation of a prognostic score to predict mortality in patients with acute-on-chronic liver failure. J Hepatol. (2014) 61:1038–47. doi: 10.1016/j.jhep.2014.06.012 24950482

[26] Moreau R Jalan R Gines P Pavesi M Angeli P Cordoba J . Acute-on-chronic liver failure is a distinct syndrome that develops in patients with acute decompensation of cirrhosis. Gastroenterology (2013) 144:1426–37.e9. doi: 10.1053/j.gastro.2013.02.042 23474284

[27] Leening MJG Vedder MM Witteman JCM Pencina MJ Steyerberg EW. Net reclassification improvement: computation, interpretation, and controversies. Ann Intern Med. (2014) 160:122–31. doi: 10.7326/M13-1522 24592497

[28] Kamran F Tang S Otles E McEvoy DS Saleh SN Gong J . Early identification of patients admitted to hospital for covid-19 at risk of clinical deterioration: model development and multisite external validation study. BMJ (2022) 376:e068576. doi: 10.1136/bmj-2021-068576 35177406 PMC8850910

[29] Gustot T Durand F Lebrec D Vincent J Moreau R. Severe sepsis in cirrhosis. Hepatology. (2009) 50:2022–33. doi: 10.1002/hep.23264 19885876

[30] McLaughlin D Shellenback L. Sepsis in patients with cirrhosis. AACN Adv Crit Care. (2016) 27:408–19. doi: 10.4037/aacnacc2016716 27959297

[31] Simonetto DA Piccolo Serafim L Gallo de Moraes A Gajic O Kamath PS. Management of sepsis in patients with cirrhosis: current evidence and practical approach. Hepatology. (2019) 70:418–28. doi: 10.1002/hep.30412 30516866

[32] Piccolo Serafim L Simonetto DA Choi DH Weister TJ Hanson AC Kamath PS . Derivation of a mortality prediction model in critical care patients with cirrhosis and sepsis. Shock. (2024) 61:382–6. doi: 10.1097/SHK.0000000000002323 38517233

[33] Chebl RB Tamim H Sadat M Qahtani S Dabbagh T Arabi YM. Outcomes of septic cirrhosis patients admitted to the intensive care unit. Medicine (Baltimore). (2021) 100:e27593. doi: 10.1097/MD.0000000000027593 34797280 PMC8601275

[34] Drolz A Horvatits T Rutter K Landahl F Roedl K Meersseman P . Lactate improves prediction of short-term mortality in critically ill patients with cirrhosis: a multinational study. Hepatology. (2019) 69:258–69. doi: 10.1002/hep.30151 30070381

[35] Kushimoto S Gando S Saitoh D Mayumi T Ogura H Fujishima S . The impact of body temperature abnormalities on the disease severity and outcome in patients with severe sepsis: an analysis from a multicenter, prospective survey of severe sepsis. Crit Care. (2013) 17:R271. doi: 10.1186/cc13106 24220071 PMC4057086

[36] Chen J Zhang W Yang Y. Hypothermia association with all-cause mortality in critically ill patients with sepsis based on the MIMIC-IV database. Sci Rep. (2025) 15:44902. doi: 10.1038/s41598-025-29166-0 41462525 PMC12749849

[37] Bhavani SV Spicer A Sinha P Malik A Lopez-Espina C Schmalz L . Distinct immune profiles and clinical outcomes in sepsis subphenotypes based on temperature trajectories. Intensive Care Med. (2024) 50:2094–104. doi: 10.1007/s00134-024-07669-0 39382693 PMC12674214

[38] Bone RC Balk RA Cerra FB Dellinger RP Fein AM Knaus WA . Definitions for sepsis and organ failure and guidelines for the use of innovative therapies in sepsis. Chest. (1992) 101:1644–55. doi: 10.1378/chest.101.6.1644 1303622

[39] Philips CA Ahamed R Rajesh S George T Mohanan M Augustine P. Update on diagnosis and management of sepsis in cirrhosis: current advances. World J Hepatol. (2020) 12:451–74. doi: 10.4254/wjh.v12.i8.451 32952873 PMC7475781

[40] Ning Y-L Li W-J Lu X Zhang Y Zhang J-W Zhou J-H. Association between heart rate and mortality in patients with septic shock: an analysis revealed by time series data. BMC Infect Dis. (2024) 24:1088. doi: 10.1186/s12879-024-10004-z 39354354 PMC11446028

[41] Kim JH Jun BG Lee M Lee HA Kim TS Heo JW . Reappraisal of sepsis-3 and CLIF-SOFA as predictors of mortality in patients with cirrhosis and infection presenting to the emergency department: a multicenter study. Clin Mol Hepatol. (2022) 28:540–52. doi: 10.3350/cmh.2021.0169 35526859 PMC9293608

[42] Ibarz M Haas LEM Ceccato A Artigas A. The critically ill older patient with sepsis: a narrative review. Ann Intensive Care. (2024) 14:6. doi: 10.1186/s13613-023-01233-7 38200360 PMC10781658

[43] Lominchar PL Orue-Echebarria MI Martín L Lisbona CJ Salcedo MM Olmedilla L . Cirrhotic patients and older people. World J Hepatol. (2019) 11:663–77. doi: 10.4254/wjh.v11.i9.663 31598193 PMC6783401

