# Supplementary material for: Development and validation of a prediction model for in-hospital mortality in intensive care unit patients with cirrhosis and sepsis: a multicentre retrospective cohort study
Source: Front Med (Lausanne). 2026 Feb 9;13:1759988. doi: 10.3389/fmed.2026.1759988 (PMC12926366; doi:10.3389/fmed.2026.1759988)
Supplement: Supplementary file 1 [file Table_1.docx]

**Supplementary Materials**

**Supplementary Table 1. ICD codes for cirrhosis.**

| **ICD Code** | **Version** | **Disease Name** |
| --- | --- | --- |
| 571.2 | ICD-9 | Alcoholic cirrhosis of liver |
| 571.5 | ICD-9 | Cirrhosis of liver without mention of alcohol |
| 571.6 | ICD-9 | Biliary cirrhosis |
| K70.31 | ICD-10 | Alcoholic cirrhosis of liver with ascites |
| K70.30 | ICD-10 | Alcoholic cirrhosis of liver without ascites |
| K74.3 | ICD-10 | Primary biliary cirrhosis |
| K74.4 | ICD-10 | Secondary biliary cirrhosis |
| K74.5 | ICD-10 | Biliary cirrhosis, unspecified |
| K74.69 | ICD-10 | Other cirrhosis of liver |
| K74.60 | ICD-10 | Unspecified cirrhosis of liver |

**Supplementary Table 2. Patient characteristics of the eICU-CRD validation set.**

| **Variable** | **Overall (n=657)** | **Survival (n=487)** | **Death (n=170)** | **P value** |
| --- | --- | --- | --- | --- |
| Age, years | 59.0 [52.0, 65.0] | 59.0 [52.0, 65.0] | 58.0 [52.0, 63.0] | 0.211 |
| Sex, male | 405 (61.6) | 298 (61.2) | 107 (62.9) | 0.755 |
| BMI, kg/m² | 27.6 [23.7, 33.2] | 27.5 [23.5, 33.3] | 27.7 [24.5, 32.8] | 0.677 |
| Emergency admission | 293 (44.6) | 227 (46.6) | 66 (38.8) | 0.095 |
| Mean arterial pressure, mmHg | 71.0 [66.0, 78.0] | 73.0 [66.0, 80.0] | 68.5 [63.2, 73.0] | <0.001 |
| Heart rate, beats/min | 90.0 [79.0, 102.0] | 88.0 [77.0, 100.0] | 98.0 [84.0, 107.9] | <0.001 |
| Respiratory rate, breaths/min | 20.0 [17.0, 23.0] | 20.0 [17.0, 22.0] | 21.8 [19.0, 25.0] | <0.001 |
| Body temperature, °C | 36.8 [36.5, 37.1] | 36.8 [36.5, 37.1] | 36.7 [36.3, 37.0] | 0.007 |
| SpO₂, % | 97.0 [96.0, 98.0] | 97.2 [96.0, 98.0] | 96.7 [95.7, 98.0] | <0.001 |
| Urine output <400 mL | 108 (16.4) | 57 (11.7) | 51 (30.0) | <0.001 |
| GCS score | 14.0 [9.0, 15.0] | 14.0 [10.0, 15.0] | 11.0 [7.0, 15.0] | <0.001 |
| Total bilirubin, umol/L | 51.3 [22.2, 107.7] | 41.0 [20.5, 85.5] | 87.0 [41.9, 160.7] | <0.001 |
| Albumin, g/L | 22.0 [19.0, 26.0] | 23.0 [19.0, 26.0] | 20.0 [16.0, 24.0] | <0.001 |
| Lactate, mmol/L | 3.3 [2.1, 5.1] | 3.1 [2.0, 4.4] | 4.7 [2.8, 7.7] | <0.001 |
| Creatinine, umol/L | 152.0 [97.2, 283.8] | 139.7 [88.4, 259.5] | 183.0 [128.4, 333.0] | <0.001 |
| WBC, ×10⁹/L | 12.6 [8.1, 19.0] | 12.0 [7.9, 17.9] | 15.8 [9.3, 24.3] | <0.001 |
| Sodium, mmol/L | 134.0 [130.0, 138.0] | 134.0 [130.0, 138.0] | 133.5 [130.0, 137.8] | 0.294 |
| International normalized ratio | 1.7 [1.4, 2.4] | 1.6 [1.3, 2.1] | 2.3 [1.7, 3.0] | <0.001 |
| IMV | 305 (46.4) | 198 (40.7) | 107 (62.9) | <0.001 |
| Vasopressor | 66 (10.0) | 42 ( 8.6) | 24 (14.1) | 0.057 |
| RRT | 18 ( 2.7) | 16 ( 3.3) | 2 ( 1.2) | 0.239 |
| Alcohol abuse history | 79 (12.0) | 60 (12.3) | 19 (11.2) | 0.797 |
| Viral hepatitis history | 48 ( 7.3) | 35 ( 7.2) | 13 ( 7.6) | 0.978 |
| Presence of ascites | 184 (28.0) | 126 (25.9) | 58 (34.1) | 0.050 |
| Hepatic encephalopathy | 126 (19.2) | 78 (16.0) | 48 (28.2) | 0.001 |
| MELD | 23.0 [16.0, 31.0] | 21.0 [15.0, 28.0] | 30.0 [21.0, 38.0] | <0.001 |
| MELD-Na | 26.0 [18.0, 32.0] | 24.0 [17.0, 30.0] | 32.0 [23.2, 38.0] | <0.001 |
| SOFA | 9.0 [6.0, 11.0] | 8.0 [6.0, 10.0] | 11.0 [8.0, 14.0] | <0.001 |
| Albumin-bilirubin | -0.7 [-1.2, -0.3] | -0.8 [-1.3, -0.4] | -0.4 [-0.9, -0.1] | <0.001 |
| Child-Pugh | 9.0 [7.0, 11.0] | 9.0 [7.0, 10.0] | 11.0 [9.0, 11.0] | <0.001 |
| CLIF-OF | 9.0 [8.0, 11.0] | 9.0 [8.0, 10.0] | 11.0 [9.0, 12.8] | <0.001 |
| CLIF-C ACLF | 50.0 [43.0, 56.0] | 48.0 [42.0, 54.0] | 56.0 [50.0, 62.0] | <0.001 |
| CLIF-SOFA | 8.0 [6.0, 10.0] | 7.0 [5.0, 9.0] | 10.0 [8.0, 12.0] | <0.001 |

**Supplementary Table 3. Patient characteristics of the SZPH-ICU validation set.**

| **Variable** | **Overall (n=131)** | **Survival (n=81)** | **Death (n=50)** | **P value** |
| --- | --- | --- | --- | --- |
| Age, years | 61.0 [50.0, 72.5] | 60.0 [48.0, 71.0] | 66.0 [54.8, 74.0] | 0.139 |
| Sex, male | 98 ( 74.8) | 59 ( 72.8) | 39 ( 78.0) | 0.650 |
| BMI, kg/m² | 23.3 [22.6, 23.9] | 22.9 [22.1, 23.6] | 23.6 [23.2, 24.4] | <0.001 |
| Emergency admission | 45 ( 34.4) | 24 ( 29.6) | 21 ( 42.0) | 0.208 |
| Mean arterial pressure, mmHg | 84.0 [76.0, 90.5] | 85.0 [78.0, 90.0] | 82.0 [73.0, 92.0] | 0.455 |
| Heart rate, beats/min | 98.0 [87.0, 108.0] | 94.0 [87.0, 107.0] | 101.5 [89.5, 111.8] | 0.059 |
| Respiratory rate, breaths/min | 19.0 [16.0, 23.0] | 18.0 [16.0, 22.0] | 22.0 [17.0, 23.0] | 0.032 |
| Body temperature, °C | 37.0 [36.0, 37.0] | 37.0 [37.0, 37.0] | 37.0 [36.0, 37.0] | 0.268 |
| SpO₂, % | 98.0 [97.0, 99.0] | 98.0 [97.0, 99.0] | 98.0 [97.0, 99.0] | 0.137 |
| Urine output <400 mL | 23 ( 17.6) | 9 ( 11.1) | 14 ( 28.0) | 0.026 |
| GCS score | 15.0 [13.0, 15.0] | 15.0 [13.0, 15.0] | 14.5 [13.0, 15.0] | 0.508 |
| Total bilirubin, umol/L | 52.3 [29.4, 126.1] | 44.3 [25.9, 70.9] | 79.3 [43.6, 203.2] | 0.002 |
| Albumin, g/L | 28.6 [25.6, 31.3] | 28.6 [25.4, 31.0] | 29.0 [26.3, 31.8] | 0.334 |
| International normalized ratio | 1.7 [1.4, 2.0] | 1.6 [1.4, 1.9] | 1.8 [1.5, 3.3] | 0.001 |
| Lactate, mmol/L | 3.3 [2.0, 6.1] | 2.4 [1.7, 4.3] | 5.7 [3.2, 10.3] | <0.001 |
| Creatinine, umol/L | 127.0 [78.0, 243.5] | 106.0 [65.0, 223.0] | 168.0 [116.5, 276.3] | 0.001 |
| WBC, ×10⁹/L | 11.2 [7.2, 16.1] | 10.2 [7.4, 15.8] | 11.8 [7.1, 16.5] | 0.678 |
| Sodium, mmol/L | 134.7 [131.7, 136.6] | 134.9 [133.0, 136.8] | 133.8 [130.8, 136.2] | 0.137 |
| IMV | 56 ( 42.7) | 29 ( 35.8) | 27 ( 54.0) | 0.062 |
| Vasopressor | 64 ( 48.9) | 33 ( 40.7) | 31 ( 62.0) | 0.029 |
| RRT | 50 ( 38.2) | 25 ( 30.9) | 25 ( 50.0) | 0.045 |
| Presence of ascites | 50 ( 38.2) | 29 ( 35.8) | 21 ( 42.0) | 0.600 |
| Hepatic encephalopathy | 32 ( 24.4) | 14 ( 17.3) | 18 ( 36.0) | 0.027 |
| Viral hepatitis history | 39 ( 29.8) | 22 ( 27.2) | 17 ( 34.0) | 0.525 |
| Alcohol abuse history | 24 ( 18.3) | 15 ( 18.5) | 9 ( 18.0) | 1.000 |
| MELD | 23.0 [17.0, 33.0] | 21.0 [15.0, 26.0] | 30.0 [22.0, 38.0] | <0.001 |
| MELD-Na | 26.0 [18.0, 35.0] | 22.0 [17.0, 29.0] | 32.0 [24.0, 38.8] | <0.001 |
| SOFA | 9.0 [6.0, 12.0] | 8.0 [6.0, 10.0] | 10.0 [7.2, 12.8] | 0.001 |
| Albumin-bilirubin | -1.2 [-1.5, -0.9] | -1.2 [-1.6, -1.0] | -1.2 [-1.4, -0.9] | 0.353 |
| Child-Pugh | 9.0 [7.0, 10.0] | 9.0 [7.0, 10.0] | 10.0 [8.2, 11.0] | 0.001 |
| CLIF-OF | 10.0 [8.0, 12.0] | 9.0 [7.0, 10.0] | 11.0 [9.0, 13.8] | <0.001 |
| CLIF-C ACLF | 51.0 [45.0, 59.0] | 49.0 [42.0, 54.0] | 58.0 [49.2, 63.5] | <0.001 |
| CLIF-SOFA | 9.0 [6.0, 12.0] | 7.0 [5.0, 10.0] | 11.0 [9.0, 14.0] | <0.001 |
